# Glutathione Deficiency in Cardiac Patients Is Related to the Functional Status and Structural Cardiac Abnormalities

**DOI:** 10.1371/journal.pone.0004871

**Published:** 2009-03-25

**Authors:** Thibaud Damy, Matthias Kirsch, Lara Khouzami, Philippe Caramelle, Philippe Le Corvoisier, Françoise Roudot-Thoraval, Jean-Luc Dubois-Randé, Luc Hittinger, Catherine Pavoine, Françoise Pecker

**Affiliations:** 1 AP-HP, Groupe hospitalier Henri-Mondor Albert-Chenevier, Fédération de Cardiologie, Département de Chirurgie Cardiaque, Créteil, France; 2 INSERM, U955, Créteil, France; 3 Université Paris12, Faculté de Médecine, UMR-S955, Créteil, France; 4 INSERM, Centre d'Investigation Clinique 006, Créteil, France; 5 Platform of biological resources, Groupe hospitalier Henri-Mondor Albert-Chenevier, Créteil, France; 6 AP-HP, Groupe hospitalier Henri-Mondor Albert-Chenevier, Département de Recherche Clinique- Santé Publique, Créteil, France; Instituto de Química, Universidade de São Paulo, Brazil

## Abstract

**Background:**

The tripeptide glutathione (L-gamma-glutamyl-cysteinyl-glycine) is essential to cell survival, and deficiency in cardiac and systemic glutathione relates to heart failure progression and cardiac remodelling in animal models. Accordingly, we investigated cardiac and blood glutathione levels in patients of different functional classes and with different structural heart diseases.

**Methods:**

Glutathione was measured using standard enzymatic recycling method in venous blood samples obtained from 91 individuals, including 15 healthy volunteers and 76 patients of New York Heart Association (NYHA) functional class I to IV, undergoing cardiac surgery for coronary artery disease, aortic stenosis or terminal cardiomyopathy. Glutathione was also quantified in right atrial appendages obtained at the time of surgery.

**Results:**

In atrial tissue, glutathione was severely depleted (−58%) in NYHA class IV patients compared to NYHA class I patients (P = 0.002). In patients with coronary artery disease, this depletion was related to the severity of left ventricular dysfunction (P = 0.006). Compared to healthy controls, blood glutathione was decreased by 21% in NYHA class I patients with structural cardiac disease (P<0.01), and by 40% in symptomatic patients of NYHA class II to IV (P<0.0001). According to the functional NYHA class, significant depletion in blood glutathione occurred before detectable elevation in blood sTNFR1, a marker of symptomatic heart failure severity, as shown by the exponential relationship between these two parameters in the whole cohort of patients (r = 0.88).

**Conclusions:**

This study provides evidence that cardiac and systemic glutathione deficiency is related to the functional status and structural cardiac abnormalities of patients with cardiac diseases. These data also suggest that blood glutathione test may be an interesting new biomarker to detect asymptomatic patients with structural cardiac abnormalities.

## Introduction

Despite considerable advances in treatment, heart failure remains associated with high morbidity and mortality worldwide [1]–[3]. Better identification of asymptomatic individuals with structural cardiac abnormalities would improve outcomes and reduce incidence of heart failure.

The pro-inflammatory cytokine tumor necrosis factor-alpha (TNF) and the cleaved extracellular domain of its type-1 receptor (sTNFR1) are recognized biomarkers of heart failure severity and adverse outcomes of the disease [4]–[11]. B-type natriuretic peptide (BNP) and the amino-terminal fragment of its precursor hormone (NT-pro-BNP) secreted in response to myocardial stress have also received considerable attention as potential screening and prognostic tests for symptomatic, New York Heart Association (NYHA) class II to IV patients [12]–[16]. However, neither TNF nor sTNFR1 or BNP peptides do help to the screening of asymptomatic patients suspected of having a structural heart disease. Only very recently, circulating MMP-9 has been associated with cardiovascular risk factors in middle-aged normal population [17].

Exacerbated TNF and sTNFR1 expression is related to systemic and cardiac glutathione deficiency in animal models of heart failure [18], [19], and in advanced heart failing patients [19], [20]. In fact, the antioxidant tripeptide glutathione (L-gamma-glutamyl–cysteinyl–glycine) is essential for vascular and cardiac function [19], [20], and determines cell survival [21], [22].

We hypothesized that functional status and cardiac structural remodelling of patients were related to glutathione deficiency. The purpose of the present study was to explore the glutathione levels in cardiac tissue and blood of patients with cardiac structural abnormalities in relation to NYHA functional classification, left ventricular ejection fraction (LVEF) and blood sTNFR1.

## Methods

### Patients

The study included 76 patients undergoing cardiac surgery (coronary artery bypass grafting, aortic valve replacement, orthotopic heart transplantation and ventricular assist device implantation) from 2004 to 2007. Clinical data and transthoracic echocardiographies (Vivid 7, GE, Norway), using american society of echocardiography recommendations [23], were obtained for all individuals. To distinguish patients with systolic LV dysfunction from those with preserved LV function, we used as cut-off value 45% LVEF, which is the mean of the 40–50% range proposed by the new ESC guidelines [24]. Permanent atrial fibrillation was defined as long standing atrial fibrillation in which cardioversion had failed or had been foregone, according to the ESC guidelines [25]. Venous blood samples and right atrial appendages were obtained from patients undergoing cardiac surgery for coronary artery bypass graft or aortic valve replacement with cardiopulmonary bypass. Blood samples only were obtained from patients undergoing left ventricular assist device implantation. Right atrial specimen and 2 venous blood samples were taken into cryotubes at initiation of cardiopulmonary bypass, immediately frozen in liquid nitrogen and stored at −80°C until use. Patients with sepsis, endocarditis, renal failure or impaired liver function were excluded.

Fifteen healthy volunteers were recruited by the Centre d'Investigation Clinique of the Hôpital Henri Mondor. Blood samples obtained from each fasting volunteer, clinical data, and transthoracic echocardiographies were processed as described above for patients. Volunteers included 8 males and 7 females with mean age of 52±4 years (range: 30–70 years), mean LVEF of 61.6±1.4% (range: 55–68%), mean blood glutathione level of 2.13±0.4 (range: 1.84–2.36 mM) and mean blood sTNFR1 level of 0.25±0.01 ng/ml (range: 0.20–0.33 ng/ml).

All patients had given written informed consent before surgical procedures were performed. All studies are conformed to the Declaration of Helsinki and were approved by our institutional ethics committee (AP-HP, Groupe hospitalier Henri-Mondor Albert-Chenevier, Créteil, F-94010, France).

### Assays for glutathione and sTNFR1

Atrial tissue samples were cut into 20 µm sections. Homogenates were prepared from 5 frozen sections of each sample by homogenization at 4°C, in 200 µl of 50 mM Hepes, pH 7.4, containing protease inhibitors (1 mM PMSF, 2 µg/ ml leupeptin, 2 µg/ ml aprotinin), using a Qiagen TissueLyzer.

Glutathione was measured in atrial homogenates or whole blood according to a modification of Tietze's recycling assay [26] as previously used [18] and as thoroughly described by Rahman et al. [27]. In short, it is a spectrophotometric/microplate reader assay method, relying on oxidation of reduced glutathione (GSH) by the sulfhydryl reagent 5,5′-dithio-bis(2-nitrobenzoic acid) (DTNB) to form the yellow derivative 5′-thio-2-nitrobenzoic acid (TNB), measurable at 405 nm. Glutathione disulfide (GSSG) is recycled to GSH by glutathione reductase in the presence of NADPH. This method is simple, convenient, sensitive, accurate and rapid, and can assay glutathione in whole blood and tissues. In addition, it uses sulfosalicylic acid for sample preparation, which inhibits gamma-glutamyl transferase and limits glutathione loss [27].

sTNFR1 was quantified in whole blood with ELISA kits (Quantikine, R&D Systems).

### Statistical analysis

Results are given as means±sem. Continuous data were analyzed by Mann-Whitney test or Kruskal-Wallis test combined with Dunn post-test, as appropriate (Prism, GraphPad Software Inc). Discontinuous data were analyzed using a Chi square test. Differences were considered statistically significant at P<0.05 (two tailed). We determined the cut-off values of blood glutathione and blood sTNFR1 level to discriminate cardiac patients (NYHA class I to IV) from healthy controls by constructing receiver operating characteristics (ROC) curves relating each marker to NYHA class. Areas under the ROC curves (AUROCs) are given with their 95% confidence intervals. Cut-off values were chosen to optimize the couple of values sensitivity/specificity.

## Results

### Clinical and biological characteristics of the patients

**Figure 1:**
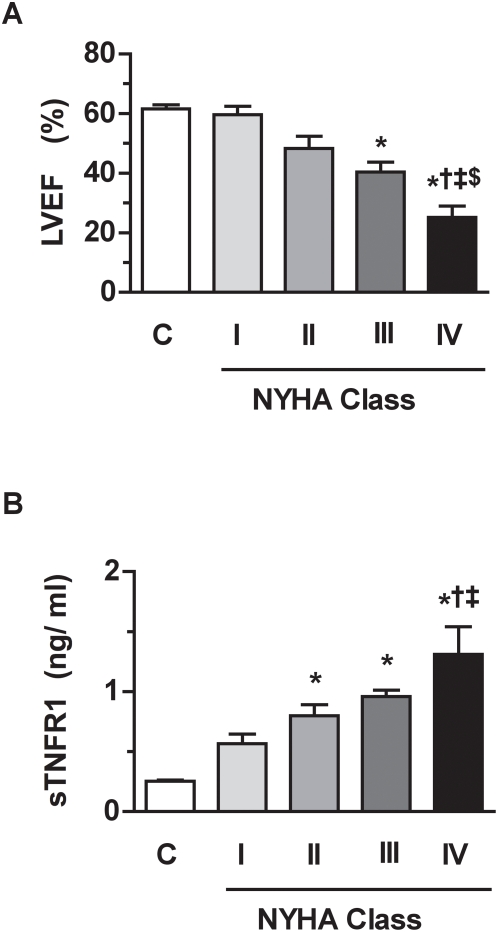
Relations between LVEF or blood sTNFR1 level and NYHA class in cardiac patients and healthy controls. The cohort displayed standard relations between functional NYHA class and LVEF (A) or blood sTNFR1 level (B). LVEF: left ventricular ejection fraction. sTNFR1: cleaved extracellular domain of TNFR1. Linear trends P<0.0001. *P<0.05 vs healthy controls (C). †P<0.05 vs NYHA class I; ‡P<0.05 vs NYHA class II; $P<0.05 vs NYHA class III.

The clinical and biological characteristics of the 76 patients undergoing surgery for dilated cardiomyopathy (transplantation or mechanical assist device implantation; n = 8), aortic valve stenosis (AS; n = 25) or coronary artery disease (CAD; n = 43) are reported in **Table 1**. Patients with CAD and patients with AS constituted the two principal groups of our cohort. The three groups had cardiac structural abnormalities. Patients with CAD and patients with dilated cardiomyopathy had reduced LVEF while AS patients displayed significant hypertrophy of septal (ST) and posterior (PWT) end-diastolic walls but preserved LVEF (**Table 1**). In the cohort, 22% of the patients were of functional NYHA class I, 31% of NYHA class II, 29% of NYHA class III and 18% of NYHA class IV. As compared to healthy controls (mean LVEF of 62±1% and mean age of 52±4 years), patients of NYHA class I had a preserved LVEF (**Fig. 1A**). In patients of NYHA class II to IV, LVEF declined progressively, but only patients of NYHA class III and IV had statistically depressed LVEF, with mean values approaching 40±3% and 25±4%, respectively (**Fig. 1A**), related to a 3- to 5-fold elevation in blood sTNFR1 level as compared to healthy controls (**Fig. 1B**). Of note, mean blood sTNFR1 level in NYHA class I patients was not statistically different from that of healthy controls (**Fig. 1B**).

**Table 1:** Baseline characteristics of patients undergoing cardiac surgery.

Parameters		All patients	CAD	AS	P-value
**Demographic**					
	Male/ female (n/ n)	60/ 16	37/ 6	15/ 10	0.01
	Age (years)	66±1	62±2	75±2	<0.0001
	NYHA (mean)	2.3±0.1	2.2±0.2	2.6±0.2	NS
	NYHA class I (%)	22	31	5	
	NYHA class II (%)	31	31	42	
	NYHA class III (%)	29	26	37	
	NYHA class IV (%)	18	12	16	
**Clinical**					
	Hypertension (%)	56	64	36	NS
	Hypercholesterolemia (%)	56	81	47	0.03
	Diabetes mellitus (%)	35	45	17	NS
	Permanent atrial fibrillation (%)	12	5	24	0.01
**Echocardiographic**					
	LVEF (%)	45±2	45±2	53±4	0.06
	LVEDD (mm)	54±1	55±2	50±2	0.04
	iLVEDD (mm.cm^−2)^	30±1	30±1	29±1	NS
	ST (mm)	11.6±0.4	10.3±0.4	13.3±0.7	0.0001
	PWT (mm)	10.8±0.4	9.8±0.4	12.1±0.6	0.002
	LA diameter (mm)	41±2	41±2	42±3	NS
	Systolic PAP (mm Hg)	44±2	42±4	43±3	NS
**Medication**					
	Beta-blockers (%)	51	71	22	0.0005
	ACE inhibitors (%)	35	50	11	NS
	AT-II type 1 R antagonists (%)	21	20	22	NS
	Diuretics (%)	48	43	57	NS
	Aldosterone antagonists (%)	16	20	9	NS
	Statin (%)	60	77	35	0.001
**Surgical**					
	Not urgent surgery, n(%)	83	81	100	0.02
**Biochemical**					
	CRP (mg/ l)	11±2	11±3	11±6	NS
	Haemoglobin (g/ dl)	13.1±0.2	13.3±0.3	12.8±0.5	NS
	Total bilirubin (mg/ dl)	17±2	18±3	17±3	NS
	Creatinine (µmol/ l)	106±5	109±8	100±6	NS

CAD: patients with coronary artery diseases; AS: patients with aortic valve stenosis; LVEF: left ventricular ejection fraction; LVEDD: left ventricular end diastolic diameter; iLVEDD: indexed LVEDD; ST: end-diastolic septal wall thickness; PWT: end-diastolic posterior wall thickness; LAD: left atrial diameter; PAP: pulmonary artery pressure; CRP: C-reactive protein. In CAD and AS, blood glutathione was not correlated with the age (p = 0.46). Data are given as mean or percentage ±sem. P-values refer to the comparisons between CAD and AS patients.

### Right atrial glutathione in patients with cardiac disease

**Figure 2:**
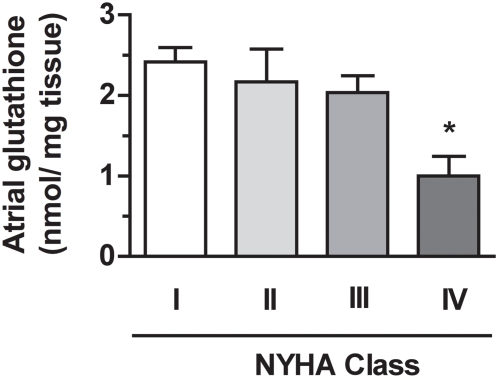
Relation between atrial tissue glutathione content and NYHA class in cardiac patients. Atrial tissue glutathione content was significantly decreased in symptomatic patients of NYHA class IV compared to asymptomatic patients of NYHA class I. *P<0.05 vs NYHA class I.

Compared to patients of NYHA class I, patients of NYHA class II and III displayed quite preserved right atrial glutathione content (**Fig. 2**). In contrast, patients of NYHA class IV demonstrated a dramatic 58% depletion in atrial tissue glutathione, dropping to 1.0±0.2 nmol glutathione/mg tissue to be compared to 2.4±0.2 nmol glutathione/mg tissue in NYHA class I patients (P = 0.002) (**Fig. 2**). Of note, as previously pointed out by Carnes et al [28], patients with permanent atrial fibrillation displayed a significant decrease in atrial glutathione content compared to patients with sinus rhythm (1.1±0.3 *vs* 2.1±0.2 nmol/ mg tissue, respectively; P<0.05) (**Table 1**).

**Figure 3:**
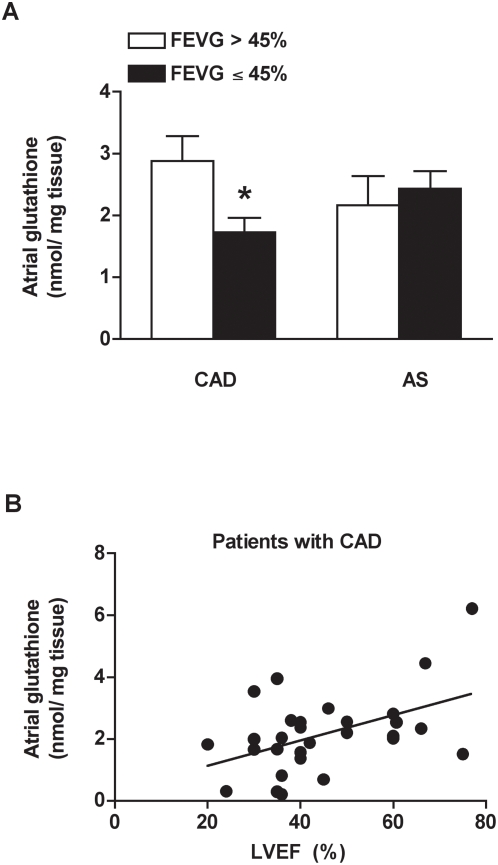
Atrial tissue glutathione content in patients with coronary artery diseases (CAD) or aortic stenosis (AS), according to preserved LVEF (>45%) or depressed LVEF (≤45%). Patients with permanent atrial fibrillation were excluded. (A) Deficiency in atrial tissue glutathione was related to LV dysfunction in CAD patients. In contrast, atrial glutathione was rather low in AS patients, independently of the LVEF value. *P<0.05 vs LVEF>45%. (B) In CAD patients, atrial tissue glutathione content correlated significantly with the LVEF value (r = 0.45, P = 0.006).

Next, we considered separately the two principal CAD and AS groups of patients, redistributed into 2 subgroups according to their LV function, after excluding patients with permanent atrial fibrillation. The subgroup of CAD patients with LVEF decline (≤45%) displayed 40% deficiency in atrial glutathione content compared to the subgroup having preserved LVEF (>45%) (1.72±0.2 vs 2.9±0.4 nmol glutathione/mg tissue, respectively; **Fig. 3A**). Indeed, atrial glutathione in CAD patients was positively correlated with LVEF (r = 0.48, P = 0.007) (**Fig. 3B**). In contrast, atrial glutathione content in AS patients was rather low, independently of the LV function (2.2±0.5 and 2.4±0.3 nmol glutathione/ mg tissue for >45% and ≤45% LVEF, respectively; **Fig. 3A**).

### Blood glutathione deficiency in patients with cardiac disease

**Figure 4:**
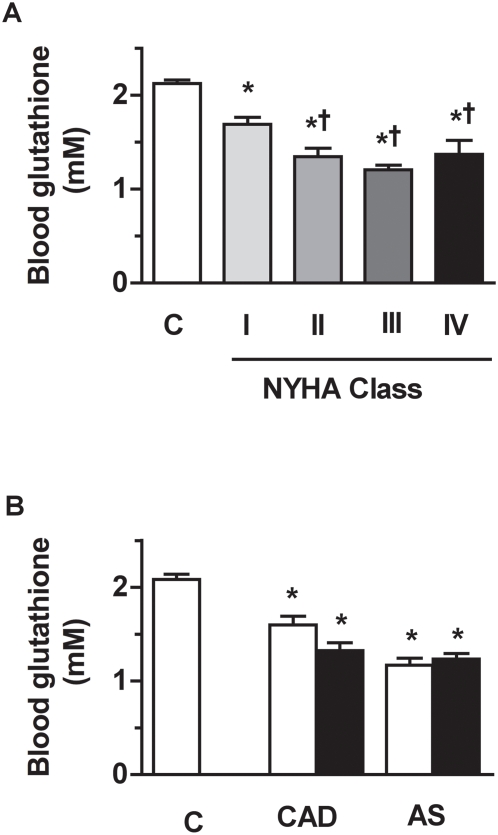
Blood glutathione level in cardiac patients and in the subgroups of patients with coronary artery diseases (CAD) or aortic stenosis (AS). (A) In patients undergoing cardiac surgery, the decrease in blood glutathione level was related to NYHA class (linear trend P<0.0001). (B) Compared to healthy controls, blood glutathione level in the CAD and AS subgroups of patients was depleted, independently of the LVEF value. *P<0.05 vs healthy controls (C); †P<0.05 vs NYHA class I.

**Figure 5:**
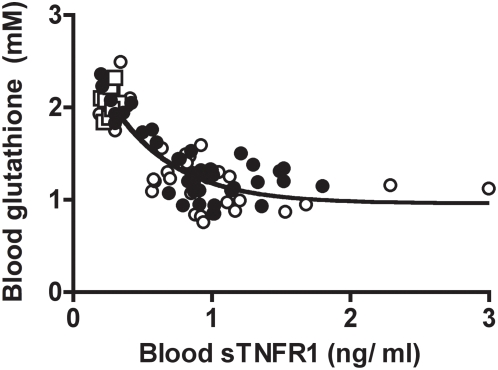
Correlation between blood glutathione and blood sTNFR1 levels in cardiac patients and healthy controls. Blood glutathione level decrease was exponentially correlated with elevation in blood sTNFR1 level in the whole cohort of patients (r = 0.88). Open squares: controls; open circles: younger patients ≤65 years (mean age = 55±1 years; r = 0.88); closed circles: older patients >65 years (mean age = 74±1 years; r = 0.87).

Compared to healthy controls, patients of NYHA class I displayed a significant 21% decrease in blood glutathione (P<0.0001) (**Fig. 4A**). Compared to patients of NYHA class I, patients of NYHA class II to IV displayed larger depletion in blood glutathione (P = 0.005) with a mean 40% decrease below the control value (P<0.0001). When considering separately the two CAD and AS groups of patients, blood glutathione level was found significantly lower than that of healthy controls, independently of the LVEF value (**Fig. 4B**).The decrease in blood glutathione was exponentially correlated with the increase in blood sTNFR1 level in the whole cohort of patients (r = 0.88; **Fig. 5**). Interestingly, significant depletion in blood glutathione occurred before detectable elevation in blood sTNFR1. To examine, whether or not ageing might influence our findings, patients were divided into two groups, younger patients (≤65 years) and older patients (>65 years). The level of blood glutathione was not related with the age of the patients, approaching 1.5±1 mM in younger patients (≤65 years, mean age of 55±1 years) and 1.4±0.1 mM in older patients (>65 years, mean age of 74±1 years). The relation between blood glutathione and sTNFR1 persisted whatever the age of the patients was (**Fig. 5**).

ROC curve analysis for discriminating NYHA class I–IV patients from healthy controls was done for each sTNFR1 and blood glutathione marker. The optimal cut-off value of sTNFR1 level was 0.33 ng/ml with an AUROC-95%CI: 0.95 [0.9–1], a sensitivity of 85.5% and a specificity of 100%. The optimal cut-off value of blood glutathione level was 1.835 mM with an AUROC-95%CI: 0.94 [0.89–0.99], a sensitivity of 81.5% and a specificity of 100%. Using these thresholds, the composite index {glutathione<1.835 mM or sTNFR1≥0.33 nmol/ml} *vs* {glutathione≥1.835 mM and sTNFR1<0.33 nmol/ml} discriminated between patients and controls with an improved sensitivity of 88.7% and a conserved 100% specificity. This result confirms the complementarity between both markers for discriminating patients from healthy controls. In fact, blood glutathione decrease allowed identification of NYHA class I patients from controls, whereas a large increase in blood sTNFR1 characterized patients of NYHA class IV.

## Discussion

The new findings of our study are twofold. Firstly, asymptomatic patients of NYHA I class display significantly deficiency in blood glutathione level compared to healthy controls. Blood glutathione deficiency worsens in patients of NYHA class II to IV in relation to blood sTNFR1 elevation, a marker of heart failure. Secondly, glutathione content in the atrial tissue is diminished by 58% in NYHA class IV patients compared to NYHA class I patients, and the degree of its decrease in CAD patients correlates with LVEF decline.

To our knowledge, our study is the first to show that blood glutathione deficiency correlates with the severity of heart failure symptoms in patients. Oxidative stress is a recognized contributor to heart failure progression [29], [30], and previous studies have pointed out changes in the redox status of glutathione in the failing heart. However, they have overlooked a possible deficiency in total glutathione content [31]. The originality of the present study was to investigate this issue in a context of identifying a possible marker of cardiac disease severity with possible implication in clinical stratification.

sTNF and its receptors sTNFR1 and sTNFR2 are pro-inflammatory molecules, the blood levels of which are associated with oxidative stress and are predictive of heart failure adverse outcomes [4], [6], [9], [11]. In keeping with previous reports, we observe a progressive increase in blood sTNFR1 level with increasing NYHA class in our patients. Blood sTNFR1 was inversely and exponentially correlated with blood glutathione, illustrating the early decrease in systemic glutathione in the course of the cardiac disease. One may also note that deficiency in systemic glutathione in cardiac patients occurs well before the drop in cardiac tissue glutathione. Several studies provide evidence for a close relationship between blood glutathione decrease and the pathogenesis of different inflammatory chronic diseases [22], [32]. In fact, systemic glutathione provides many tissues in the body, and its deficiency is likely to affect vital functions including resistance to oxidative stress, mitochondrial function and integrity, immune response and cell survival [33]. In the cardiac myocyte, glutathione deficiency fuels a vicious TNF/ sTNFR1/ oxidative stress/ neutral sphingomyelinase/ apoptosis cycle [18], [19], [34]–[36]. Accordingly, early deficiency in systemic glutathione is likely to contribute to the progression of the cardiac disease.

We have previously reported that LV of patients with end-stage cardiomyopathies, undergoing orthotopic heart transplant or ventricular assist device, is depleted by 54% in gluthatione compared to control LV [19]. In the present study, we have used atrial appendage as a surrogate for LV tissue. We recognize that atrium does not undergo same physiological requests and stresses as the LV. However, atrial appendage is accessible in patients with a large range of symptoms and with different cardiac diseases. Interestingly atrial glutathione content is depleted by 58% in NYHA class IV patients compared with NYHA class I patients, which is a drop similar to that found in end-stage failing LV. A previous study has also reported that hemodynamic impairment in both right and left ventricles of patients with heart failure subsequent to myocardial infarction correlates with a decrease in glutathione antioxidant efficiency [31]. Accordingly in the present study, LVEF decline is associated with atrial glutathione deficiency in CAD patients (r = 0.45; P = 0.006). In contrast, AS patients display equal atrial glutathione content, independently of the LVEF value. Indeed, the latter experience early LV remodelling related to increased intra-cardiac pressures and without marked LVEF decline. Finally, in agreement with the previous observation made by Carnes et al. [28], we found a 50% decrease in atrial glutathione content in patients with permanent atrial fibrillation compared to other patients with sinus rhythm, which is also consistent with the decreased incidence of postoperative atrial fibrillation observed after intravenous supplementation with the glutathione precursor, N-acetylcysteine (NAC) [37]. Taken together, these results suggest that glutathione deficiency impinges on the whole damaged heart. They also suggest that glutathione supplementation may improve cardiac cell preservation in cardiac diseases, and be a complement to contemporary treatments.

It should be mentioned that the present study is limited by the relatively small cohort size. In addition, although our results provide some evidence that the concomitant use of blood glutathione and blood sTNFR1 tests may improve the sensitivity of cardiac patient diagnostic, further comparison between blood glutathione and biomarkers of heart failure other than sTNFR1, in particular serum BNP peptides, is needed. Indeed, this was not possible in the present study in which only frozen blood samples were available. The present study did not either examine the prognostic impact of blood glutathione.

In conclusion, although further studies are needed to verify the diagnostic and/ or predictive value of blood glutathione in heart failure as a part of a multi marker panel test, this study provides evidence that cardiac and systemic glutathione deficiency is related with the functional status and the structural cardiac abnormalities of patients with cardiac diseases. These data also encourage the development of blood glutathione test as a possible new diagnostic tool for detecting asymptomatic patients with structural cardiac abnormalities.
